# Printability, Durability, Contractility and Vascular Network Formation in 3D Bioprinted Cardiac Endothelial Cells Using Alginate–Gelatin Hydrogels

**DOI:** 10.3389/fbioe.2021.636257

**Published:** 2021-02-26

**Authors:** Christopher David Roche, Poonam Sharma, Anthony Wayne Ashton, Chris Jackson, Meilang Xue, Carmine Gentile

**Affiliations:** ^1^Northern Clinical School, Faculty of Medicine and Health, The University of Sydney, Sydney, NSW, Australia; ^2^School of Biomedical Engineering, Faculty of Engineering and IT, University of Technology Sydney, Sydney, NSW, Australia; ^3^Faculty of Health and Medicine, The University of Newcastle, Callaghan, NSW, Australia

**Keywords:** 3D bioprinting, spheroids, hydrogel, bioink, durability, printability, alginate, gelatin

## Abstract

**Background:**

3D bioprinting cardiac patches for epicardial transplantation are a promising approach for myocardial regeneration. Challenges remain such as quantifying printability, determining the ideal moment to transplant, and promoting vascularisation within bioprinted patches. We aimed to evaluate 3D bioprinted cardiac patches for printability, durability in culture, cell viability, and endothelial cell structural self-organisation into networks.

**Methods:**

We evaluated 3D-bioprinted double-layer patches using alginate/gelatine (AlgGel) hydrogels and three extrusion bioprinters (REGEMAT3D, INVIVO, BIO X). Bioink contained either neonatal mouse cardiac cell spheroids or free (not-in-spheroid) human coronary artery endothelial cells with fibroblasts, mixed with AlgGel. To test the effects on durability, some patches were bioprinted as a single layer only, cultured under minimal movement conditions or had added fibroblast-derived extracellular matrix hydrogel (AlloECM). Controls included acellular AlgGel and gelatin methacryloyl (GELMA) patches.

**Results:**

Printability was similar across bioprinters. For AlgGel compared to GELMA: resolutions were similar (200–700 μm line diameters), printing accuracy was 45 and 25%, respectively (AlgGel was 1.7x more accurate; *p* < 0.05), and shape fidelity was 92% (AlgGel) and 96% (GELMA); *p* = 0.36. For durability, AlgGel patch median survival in culture was 14 days (IQR:10–27) overall which was not significantly affected by bioprinting system or cellular content in patches. We identified three factors which reduced durability in culture: (1) bioprinting one layer depth patches (instead of two layers); (2) movement disturbance to patches in media; and (3) the addition of AlloECM to AlgGel. Cells were viable after bioprinting followed by 28 days in culture, and all BIO X-bioprinted mouse cardiac cell spheroid patches presented contractile activity starting between day 7 and 13 after bioprinting. At day 28, endothelial cells in hydrogel displayed organisation into endothelial network-like structures.

**Conclusion:**

AlgGel-based 3D bioprinted heart patches permit cardiomyocyte contractility and endothelial cell structural self-organisation. After bioprinting, a period of 2 weeks maturation in culture prior to transplantation may be optimal, allowing for a degree of tissue maturation but before many patches start to lose integrity. We quantify AlgGel printability and present novel factors which reduce AlgGel patch durability (layer number, movement, and the addition of AlloECM) and factors which had minimal effect on durability (bioprinting system and cellular patch content).

## Graphical Abstract

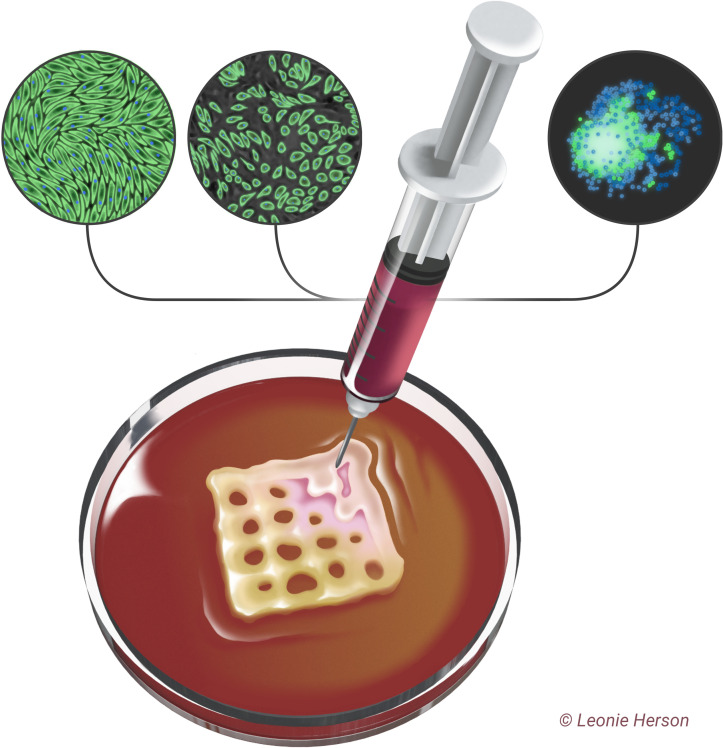

## Introduction

The latest developments in three-dimensional (3D) bioprinting technology have led to the hope that viable 3D bioprinted cardiac tissues could be generated to promote myocardial regeneration (Noor et al., 2019; Roche et al., 2020). Extrusion 3D bioprinters have been widely used as a versatile tool to deposit different cells as ‘bio-inks’ to generate complex 3D tissues, including cardiac tissues (Zhang et al., 2016; Ong et al., 2017a; Maiullari et al., 2018; Noor et al., 2019). This technology promises a safe, precise, automatable and cost-effective method to generate myocardial tissue (Noor et al., 2019; Roche et al., 2020). Extrusion 3D-bioprinters using cell-permissive pressures can extrude myocardial cells without prohibiting their ability to live, mature and function in a physiological environment (Blaeser et al., 2016). These 3D bioprinters extrude bioinks which can be made from hydrogels (Cattelan et al., 2020; Roche et al., 2020).

The bioink formulation is critical to determine printability (a function of the bioink’s rheological properties which determines how it interacts with the bioprinting process) which is important for bioprinting without damaging the end bioprinted product (Cattelan et al., 2020; Gillispie et al., 2020). After bioprinting, the tissue can be cultured to allow for a period of tissue maturation before transplantation (Roche and Gentile, 2020). During this post-printing phase, the bioink can promote tissue maturation, with durability in culture being an important characteristic to predict hydrogel disintegration (Bishop et al., 2017; Roche and Gentile, 2020), although the optimal moment to transplant after a period in culture has not previously been confirmed. During this phase, cardiomyocyte contractility should be permitted and endothelial cells within the bioprinted tissue should be permitted to organise into networks, as one of the major challenges in 3D bioprinting of cardiac tissues is the fabrication of a hierarchical vascular system within tissues (Gentile, 2016; Ong et al., 2017b; Polonchuk et al., 2017; Cui et al., 2019; Polley et al., 2020; Roche et al., 2020; Xu et al., 2020).

Here, we present a promising approach to generate 3D bioprinted cardiac patches presenting a structural endothelial cell network using alginate/gelatin (AlgGel) hydrogels – a mix of alginate (to provide an ionically cross-linkable structure for cellular patches) and gelatin (to adapt the alginate for extrusion 3D bioprinting, control rheological properties by varying the gelatin concentration and generate a bioactive hybrid hydrogel). The versatility of AlgGel hydrogels is established (Mancha Sánchez et al., 2020), balancing printability against durability characteristics in culture, whilst also permitting cardiomyocyte contractility and endothelial cell network formation. The extent to which vascular networks are able to self-assemble within patches is closely linked to several other characteristics: 1) printability (for instance, with poor printability, control of patch morphology is undermined); 2) durability (if patches disintegrate too quickly in culture then there would be no patch to host a vascular network); 3) cell viability (since survival of cells, including endothelial and other cell types, is critical); and 4) contractility must be permitted (patches which inhibit contractility of cardiomyocytes will be less suitable for co-culture with contractile cells) (Roche et al., 2020).

We hypothesised that 3D bioprinted endothelial cells can self-organise into structural vascular networks using our approach.

In testing this hypothesis, we aimed to demonstrate that even a low starting density of endothelial cells will self-organise into structural networks within 3D bioprinted patches. In this study we report on printability, durability, cell viability and endothelial cell network formation for 3D bioprinted endothelial cells in AlgGel hydrogels. Our study aims at providing new insights which may overcome common challenges in the field of bioprinting of cardiac tissues for *in vitro* and *in vivo* applications (Roche et al., 2020). The major finding of our study is that the bioprinted patches generated by using our approach present endothelial cell networks, durable structure and contractile function between 14 and 28 days in culture. Our findings have the potential to directly translate *in vitro* testing of bioprinted cardiac patches for *in vivo* applications for cardiac regeneration (Roche and Gentile, 2020).

## Materials and Methods

All procedures described in this experiment were approved by the Animal Ethics Committee at the Northern Sydney Local Health District (project number RESP17/55; 20/04/2017). Full methodological details are included in the Supplementary Materials.

### Cultures of Human Coronary Artery Endothelial Cells With Fibroblasts

Human coronary artery endothelial cells (HCAECs) (Sigma-Aldrich, MO, United States) were cultured in MesoEndo Growth Medium (Cell Applications, San Diego, CA, United States). Human dermal fibroblasts (HDFs) (Sigma-Aldrich, MO, United States) were cultured in Dulbecco’s Modified Eagle Medium (DMEM, Sigma-Aldrich, St Louis, MO, United States) with added 10% (v/v) FBS + 1% (v/v) pen/strep + 1% (v/v) L-glutamine. Cells were used for bioprinting between passage four and five.

### Vascularised Cardiac Spheroid Formation From Mouse Cardiac Cells

Mouse hearts were isolated from neonatal C57Bl/6 mice (1–5 days old), diced into 0.1–0.2 mm pieces and enzymatically digested with the Miltenyi Biotec (Bergisch Gladbach, Germany) neonatal heart dissociation kit according to the manufacturer’s instructions. Isolated cells (cardiac cell types present in a whole heart, including myocytes, endothelial cells, and fibroblasts) were suspended in DMEM + 10% (v/v) FBS + 1% (v/v) pen/strep + 1% (v/v) L-glutamine. VCSs were generated by coculturing ∼4000 mouse cardiac cells (immediately after their isolation) in 15 μl hanging drop cultures containing DMEM + 10% (v/v) FBS + 1% (v/v) pen/strep + 1% (v/v) L-glutamine, using Perfecta 3D^®^ 384-well hanging drop plates (3D Biomatrix, Ann Arbor, MI, United States). Spheroids were allowed to form for up to five days in hanging drops in a humidified incubator at 37°C with 20% (v/v) O_2_ and 5% (v/v) CO_2_. Additional complete DMEM (7 μl) was added to each hanging drop on day three. VCSs were collected and the resulting spheroid suspension was centrifuged at 300 g for 5 mins in a 50 ml Falcon tube. The resulting VCS pellet was ready for direct mixing with hydrogel to create bioink.

### Hydrogel Preparation

To prepare the alginate and gelatin (AlgGel) hydrogel 4 mg alginate and 8 mg gelatin powder was sterilised under UV light for 30 min, solubilised at 50°C in 100 ml DMEM + 10% (v/v) FBS + 1% (v/v) pen/strep + 1% (v/v) L-glut. The mixture was then either stored at 4°C or used for bioink immediately after. AlloECM hydrogel (AlloECM^®^, ROKIT, Seoul, South Korea) was prepared by adding AlloECM powder to AlgGel hydrogel at 5 and 30 mg/ml. The AlloECM-AlgGel was resuspended and mixed thoroughly in a Falcon tube at room temperature, warmed to 37°C in a water bath and then was used immediately for bioprinting. Gelatin-methacryloyl (GelMA) 10% w/v + lithium phenyl-2,4,6-trimethylbenzoylphosphinate (LAP) 0.25% (w/v) in HEPES buffer in light-blocking pneumatic 3 ml syringes was purchased (product no. IK305202, CELLINK Life Sciences, Boston, MA, United States) for use without cells as a printability and durability control hydrogel.

### Generation of Bioinks

To create bioinks for bioprinting, hydrogels were added to pellets of either mouse cardiac cell spheroids or a mixture of HCAECs and HDFs (2:1), obtained as described above. 1.5 ml prewarmed (37°C) hydrogel was added to the cell pellet by pipette, of which 0.5 ml typically produced six 10 mm^2^ patches. Each patch contained either ∼20,000 HCAECS and ∼10,000 HDFs, or ∼160,000 mouse cardiac cells (∼40 spheroids/patch). The hydrogel was resuspended until the cell pellet disappeared to ensure incorporation of most of the cells. All procedures were performed under a biological safety cabinet for the REGEMAT3D bioprinter. For the *INVIVO* and BIO X bioprinters (which have their own UV steriliser and hepafilter) this was performed within the bioprinting chamber itself.

### 3D Bioprinting

Bioprinting was performed as fully described in the Supplementary Materials (including preparation, parameter setting and bioprinting processes for the three bioprinters). AlgGel was ionically crosslinked by adding CaCl_2_ after bioprinting of all the patches in one six-well plate. GelMA was photo-crosslinked immediately after each patch layer was bioprinted by UV light photocuring.

### Printability Assessments

Printability was measured in terms of resolution, printing accuracy, shape fidelity (after 28 days in culture) and extrudability for grid pattern bioprinted patches. Resolution was assessed as the width of deposited bioink gridlines (at day one – after crosslinking immediately after bioprinting); printing accuracy was measured as the number of empty squares between bioink gridlines in the grid pattern at day one (a perfect grid should have 16 empty squares between gridlines of bioink); shape fidelity was measured as the number of empty squares remaining with time in culture at day 28; and extrudability outcome observations were (1) whether the nozzle dripped hydrogel between the bioprinting of patches (yes/no) and (2) the average number of times the nozzle was blocked requiring the nozzle to be changed per six patches bioprinted (in series one after the other). To isolate hydrogel printability outcomes for AlgGel, it was compared to GelMA using the BIO X and bioprinting with hydrogel only without cells.

### Durability Measurement

To assess survival of patches in culture, patches were monitored daily for macroscopic disintegration for up to 28 days. Culture medium was replaced every 3–4 days and the date of patch disintegration was recorded and time to disintegration (durability) analysed. To evaluate the effects of layer number on patch printability eight single-layer thickness (0.2 mm depth) acellular AlgGels were printed as controls (without cells). To evaluate the effect of minimising movement, 13 AlgGel HCAEC + HDF patches were generated and cultured under minimisation of movement conditions (slow media replacement with 1000 μl narrow-bore non-automated pipette every 7–10 days with no transfer to a microscope for observations) and these were left in culture until the first two patches in the set of 13 disintegrated. To evaluate the addition of exogenous AlloECM hydrogel to the AlgGel, 23 AlloECM + AlgGel patches were printed at low and high concentration of AlloECM. In addition, 13 acellular gelatin-methacryloyl (GelMA) patches were produced as an extended durability control as GelMA is more durable in culture.

### Cell Viability Assays, Imaging and Analysis

To assess cell survival, we evaluated cell viability in bioprinted patches after 28 days in culture by staining them with calcein-AM, ethidium homodimer (Live/Dead Assay, Invitrogen, Carlsbad, CA, United States) and Hoechst stain, used to identify live, dead and total cells (nuclei), respectively. Patches in media with stains added were incubated at 37°C for 1 h. After fresh media replacement, patches within the plate were moved to a microscope for automatic fluorescence imaging by several automated microscopic methods (using an M7000 or EVOS Fl AUTO 1 (ThermoFisher, MA, United States), Nikon Ti (Nikon, Tokyo, Japan), or IN Cell Analyzer (GE Life Sciences, IL, United States) to obtain images of the entire patches. Quantification of stained cells was performed by random grid sampling and computer-based estimation using FIJI (ImageJ) software.

### Patch Contractile Activity Evaluation

To observe contractile activity, patches were monitored by video light microscopy for intrinsic oscillations. When contractile oscillations were observed, phase contrast microscopy video recordings were obtained using an Olympus CKX53 microscope (Olympus, Tokyo, Japan), with the beating patch contrasted with non-contractile hydrogel in the same conditions. To count the rate of beating activity, videos were played in slow motion and the average rate taken from multiple samples.

### 3D Bioprinted Cell Staining, Imaging and Analysis

To evaluate endothelial cell organisation into structural networks, we stained bioprinted patches with antibodies against CD31 to identify any structural endothelial cell network-like formation after 28 days in culture. After 28 days HCAEC + HDF-containing and mouse VCS patches were first fixed and then stained using antibody against human/mouse CD31 and Hoechst stain for endothelial cells and nuclei, respectively (see Supplementary Materials for full protocol). They were imaged by light microscopy and with a confocal microscope (LSM 800, Zeiss, Oberkochen, Germany). Images were analysed with ImageJ (NIH, Bethesda, United States). For 3D rendering analysis, confocal images were processed by Imaris v7.6 (Oxford Instruments, Zurich, Switzerland).

### Statistical Analysis

Results were analysed using PRISM (GraphPad, San Diego, CA, United States). Hypothesis testing for categorical data was performed using the chi-square test. Hypothesis testing for continuous data was performed using the two-tailed Mann–Whitney U test or the Kruskal–Wallis test for a difference between two or any of three non-parametric data groups, respectively. Descriptive statistics (Kaplan–Meier survival/durability data) were tested using pairwise Log-rank (Mantel Cox) tests.

## Results

### Patch Printability

**FIGURE 1:**
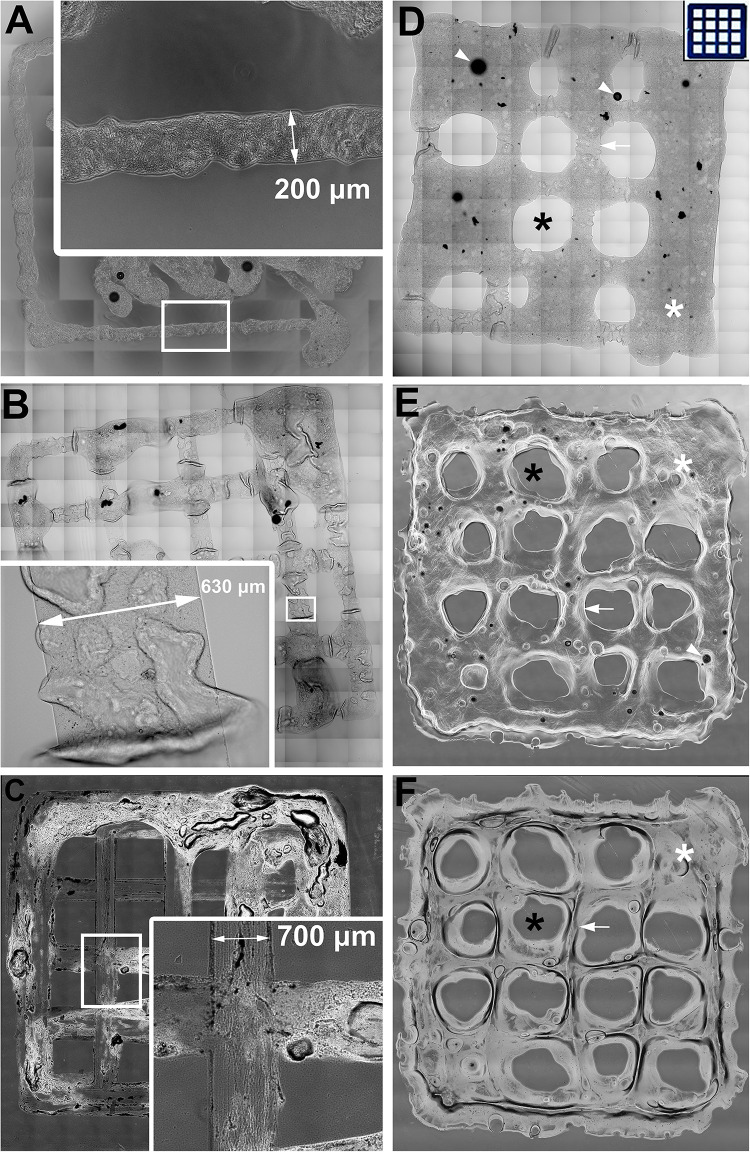
Assessing printability for extrusion 3D bioprinting with AlgGel hydrogels and GelMA. **(A–C)** Representative images of 10 mm^2^ AlgGel patches 3D bioprinted using three different bioprinters: a custom-made REGEMAT3D **(A)**, the ROKIT *INVIVO* **(B)**, and the CELLINK BIO X **(C)**. The resolution (line width) of the bioprinted grid patches was between 200 and 700 μm. Printing accuracy of AlgGel **(D)** and GelMA **(E)** was measured as the number of empty squares preserved (black asterisks) between lines of bioink (white arrow) compared to the blueprint instructions input into the software on day one **(D)** inset panel). Hydrogel filled seven of the intended 16 empty spaces in **(D)**, whereas in **(E)** two were filled (white asterisks): an example of a printing accuracy of 56 and 88% for these individually displayed representative patches, respectively. The day one GelMA patch in **(E)** is shown after 28 days in culture in **(F)**, still with two spaces filled (a shape fidelity of 100% for this displayed representative patch by this measure). The black dots (white arrowheads) in **(D)** and **(E)** are air bubbles. Scale bars not shown (all patches 1 cm^2^). Overall in the complete sample (from which these displayed representative patches are taken), printing accuracy was higher for AlgGel with 78/176 (45%) of AlgGel empty internal squares in the grid pattern preserved at day one compared to 53/208 (25%) for GelMA (*p* < 0.05; χ2 test; *n* = 384); shape fidelity after 28 days in culture was only different if it included the increased print accuracy for AlgGel on day one, with 72/176 (41%) of squares remaining preserved at day 28 for AlgGel and 51/208 (25%) for GelMA (*p* < 0.05; χ^2^ test; *n* = 384). However, correcting for differences in print accuracy on day one, the 28-day shape fidelity rate was 72/78 (92%) for AlgGel and 51/53 (96%) for GelMA (*p* = 0.36; χ^2^ test; *n* = 131).

To evaluate printability outcomes such as resolution, printing accuracy, shape fidelity and extrudability, 5 × 5 line grid patches were bioprinted instead of patches completely filled with bioink, so that these printability measures could be assessed. Patches were bioprinted with a geometry of 10 × 10 × 0.4 mm (length x width x depth) as shown in Figure 1. For all three bioprinters, printability outcomes (resolution, printing accuracy, shape fidelity and extrudability) were similar. For AlgGel hydrogel patches, resolution was similar compared to GelMA; printing accuracy was higher for AlgGel with 78/176 (45%) of AlgGel empty internal squares in the grid pattern preserved at day one compared to 53/208 (25%) for GelMA (*p* < 0.05; χ^2^ test; *n* = 384); shape fidelity after 28 days in culture was only different if it included the increased print accuracy for AlgGel on day one, with 72/176 (41%) of squares remaining preserved at day 28 for AlgGel and 51/208 (25%) for GelMA (*p* < 0.05; χ^2^ test; *n* = 384). However, correcting for differences in print accuracy on day one, the 28-day shape fidelity rate was 72/78 (92%) for AlgGel and 51/53 (96%) for GelMA (*p* = 0.36; χ^2^ test; *n* = 131); for extrudability, AlgGel dripped hydrogel from the nozzle and GelMA did not and AlgGel had zero nozzle blockages per six patches compared to one per six patches for GelMA (AlgGel tended toward more flow than input software instructions and GelMA tended toward less flow). Overall, no significant difference in printability was observed between bioprinters but AlgGel had a 1.7x higher printing accuracy, presented some dripping from the nozzle and had no nozzle blockages compared to GelMA hydrogel.

### Patch Durability in Culture

**FIGURE 2:**
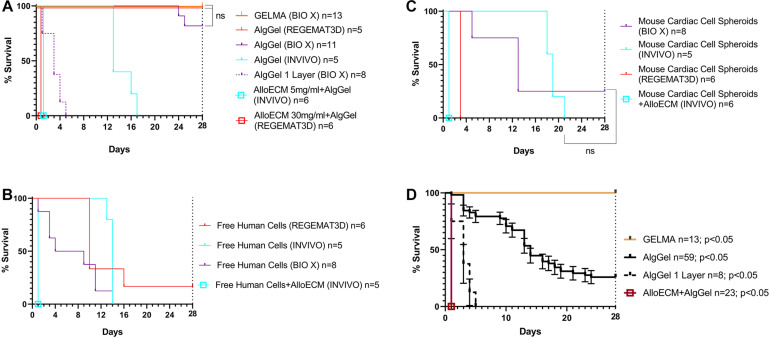
Durability assessments for 3D bioprinted patches in cell culture medium up to 28 days. **(A–C)** Survival analyses grouped by different patch cellular content, either hydrogel without cells **(A)**, with HCAECs and HDFs **(B)** or mouse cardiac cell in VCSs **(C)**. All curves are statistically significant compared to adjacent curves unless marked by “ns” and a black linking line connecting two or more similar (non-significant) curves; *p* < 0.05 Log-rank (Mantel-Cox) test of each line compared pairwise to each other line on the survival curve. In **(A)** identical curves are moved off centre to prevent complete line overlap (applies to AlloECM and GelMA curves). Each curve represents one print run of a series of patches bioprinted one after the other from the same batch of hydrogel (*n* = 5–13 per group). As cellular content had negligible influence on patch durability, pooled analysis of all patches is shown in **(D)**. Overall, these results show that survival was similar whether patches contained AlgGel alone or with cells **(A–C)**. They also show that AlgGel had a median survival of 14 days in culture overall **(D)**, which was reduced by bioprinting single layer patches instead of standard thickness (double layer) or the addition of AlloECM (fibroblast-derived extracellular matrix hydrogel). Compared to GelMA (which is more durable in culture) AlgGel presented a median survival showing that it is likely to need transplanting sooner (at 14 days in culture), because leaving patches to culture for 28 days risks many patches fragmenting and becoming unsuitable for transplantation.

To assess patch survival in culture conditions following bioprinting, patches were cultured in media at 37°C for up to 28 days to evaluate durability in the post-printing, pre-transplantation phase (Figure 2). The different bioprinting system used had little effect on the overall durability of the resulting AlgGel patches (Figures 2E,F). Overall, median (and IQR) for time in days to disintegration (durability) of AlgGel-based patches in media in six-well plates was 10 (3–19) with patches printed by the REGEMAT3D, 14 (13.5–18) with the *INVIVO*, and 14 (10–28) with the BIO X (*p* = 0.93; Kruskal–Wallis test for a difference in any of the three groups). The cellular patch content (mouse VCS, free (not in spheroid) HCAECs + HDFs or acellular hydrogel alone) also had little effect on patch durability (*p* = 0.17; Kruskal–Wallis test) (Figure 2). Kaplan–Meier survival curves suggested that the addition of cells compared to hydrogel on its own might reduce the number of bioprinting runs (sessions producing a set of patches) where all patches in that run survived to 28 days (Figures 2A–C). However, survival analyses revealed no strong pattern for durability between different cellular contents within the AlgGel patches.

As the bioprinting system and cellular content had minimal effect on patch durability, pooled analysis of all AlgGel patches was performed and median durability overall was 14 days (interquartile range (IQR) 10-27; *n* = 59; *p* < 0.05) (Figure 2D), whereas 13/13 GelMA control patches were intact at the end of 28 days (*p* < 0.05). AlgGel acellular patches with one layer were less durable (median survival 3 days; IQR 2.5–4 days; *n* = 8; *p* < 0.05) than two-layer AlgGel acellular patches (median survival 28 days; IQR 19–28; *n* = 20; *p* < 0.05) (Figure 2D). The minimisation of movement protocol allowed AlgGel patches to be cultured until day 66 before the end point of 2/13 of these patches losing their integrity (unsuitable for transplantation) was reached. The addition of AlloECM to AlgGel reduced the durability of patches: whilst AlloECM + AlgGel patches retained their structure after bioprinting, 23/23 patches disintegrated in < 1 day (Figures 2D,G). This was regardless of the AlloECM concentration in the AlgGel hydrogel (5 or 30 mg/ml), either in the presence or absence of cellular content (Figures 2A–C). Overall, factors which reduced patch durability in culture were: bioprinting patches with only one layer of depth, movement disturbance such as during media changes and transfer to a microscopy platform and the addition of AlloECM. Factors which had negligible impact on durability in culture were the bioprinting system used and the cellular content.

### Cell Viability

**FIGURE 3:**
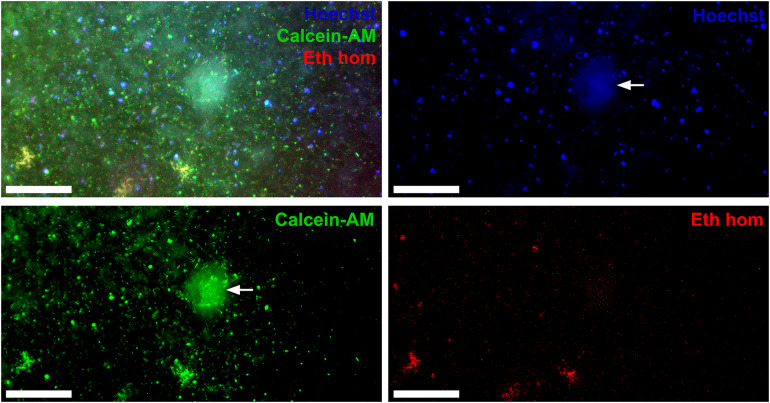
Bioprinted HCAECs and HDFs in AlgGel hydrogel are viable at 28 days. HCAEC + HDFs within a patch 3D bioprinted with the REGEMAT3D shown after 28 days in culture. This patch was stained with Hoechst (blue—nuclei), calcein-AM (green—live cell cytoplasm), ethidium homodimer (red—dead cell nuclei). Despite hydrogel autofluorescence (white arrow), the patch shows live cells (green), and nuclei (blue) at low magnification throughout this representative segment of patch. The live/dead ratio was ∼2.3 at 28 days in culture. Magnification bar = 500 μm.

**FIGURE 4:**
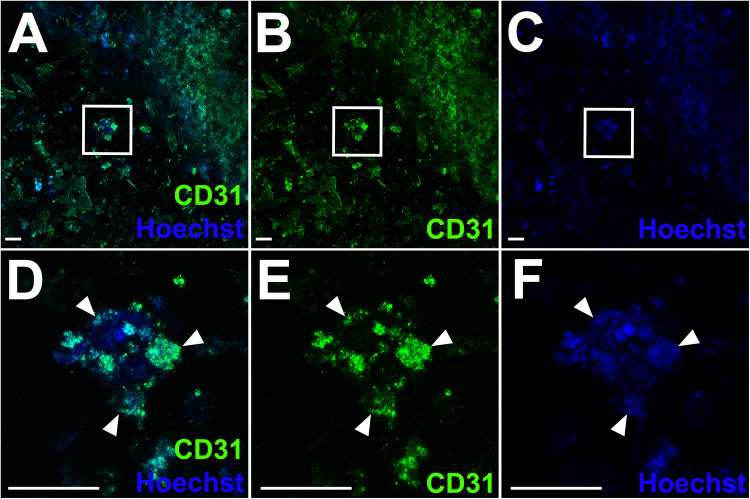
CD31-positive endothelial cells within a patch containing mouse cardiac spheroids. **(A–F)** Laser scanning confocal microscopy (LSCM) images of a BIO X-bioprinted AlgGel patch containing neonatal mouse cardiac spheroids stained for cell nuclei (blue) and CD31^+^ endothelial cells (green). Scale bars **(A–C)** = 100 μm. **(A)** Merged channel image showing CD31^+^ cells across a representative segment of patch and a spheroid still intact after bioprinting followed by 28 days in culture (inset panel). **(B)** and **(C)** show CD31 stain and Hoechst stain (nuclei), respectively. **(D)** Magnified image of the spheroid (white arrowheads) highlighted in panel **(A)**. CD31 stain and Hoechst (nuclei) are shown in **(E)** and **(F)**, respectively. Scale bars **(D–F)** = 100 μm.

To evaluate cell survival within patches at 28 days, our analysis of epifluorescence microscopy images at low magnification showed viable cells surrounded by hydrogel components of the patches which were variably autofluorescent (Figure 3). By random grid sampling, estimated live cell density was ∼80 cells/mm^2^/layer (∼16,000 cells per patch) which is a viability rate of ∼53% from the ∼30,000 cells per patch on initial bioprinting. We measured a live/dead ratio equal ∼2.3 at 28 days for these human cells. Viability in mouse mixed cardiac cell (VCS) patches (Figure 4) could not be as reliably quantified because many patch-embedded cells were in 3D spheroids. Using a software-based analysis of less autofluorescent confocal microscopy images we measured a live:dead cell ratio of 9:1 for mouse VCSs. VCS diameter was ∼150 μm at 28 days in culture as shown in Figure 4. This did not change from day one (*data not shown*), confirming also the fact that VCSs maintained their shape in culture. Overall, our method was associated with viable cells in patches, even after bioprinting and 28 days in culture.

### Patch Contractility

To assess whether patches were able to permit contractility of cardiomyocytes, throughout the 28 day experiments all mouse VCS-containing patches were evaluated under a light microscope for contractile activity (Supplementary Video 1). Five patches started to display irregular contractile activity on day seven, two on day 10 and one on day 13. Despite six out of eight patches breaking into fragments over the course of the 28 days, all eight patches (or fragments) still showed some contractile activity at the experiment end at day 28. The average rate of (non-fragmented) patch contractions was 258 beats/min (range 230–288) and the rate did not change with time in culture. Supplementary Video 1 shows VCS patch contractility in real-time and the patch floating in media can be seen oscillating compared to adjacent static areas of non-contractile hydrogel on the well floor. Altogether, the contractility observed in our neonatal mouse VCS-AlgGel patches suggests that there is no barrier in principle to generating contractile cardiac tissue using cardiac spheroids in AlgGel hydrogel (Supplementary Video 1).

### Patch Endothelial Cell Network Structural Organisation

To assess CD31^+^ endothelial cells’ ability to self-organise into structural networks, we observed for network formation in VCS-containing patches and patches with freely suspended HCAECs with HDFs, stained for CD31^+^ cells. For VCS patches, our confocal analysis showed that some CD31^+^ mouse cardiac endothelial cells in VCS remained in spheroids even after bioprinting and 28 days in culture (Figure 4D). The median length of CD31^+^ linear human endothelial cell structures was 149 μm (IQR 91–225 μm), median width was 46 μm (IQR 29–80 μm) and CD31^+^ endothelial cell covered area in the hydrogel was ∼2.7%. For these patches (which contained VCS), the CD31^+^ endothelial cells did not present extensive endothelial cell organisation into networks (Figure 4), despite some of the cells moving out of their spheroids (Figure 4D), consistent with observations in a previous study (Fleming et al., 2010). Some migrating CD31^+^ mouse cardiac endothelial cells (or clusters of these cells) were observed within the bioprinted patch outside of spheroids, suggesting that at day 28 patches contained a mixture of cells which had migrated from their spheroids into the hydrogel, cell clusters which had moved away from their initial spheroid and cells which had remained in the same spheroids present at the initial bioprinting (Figure 4).

**FIGURE 5:**
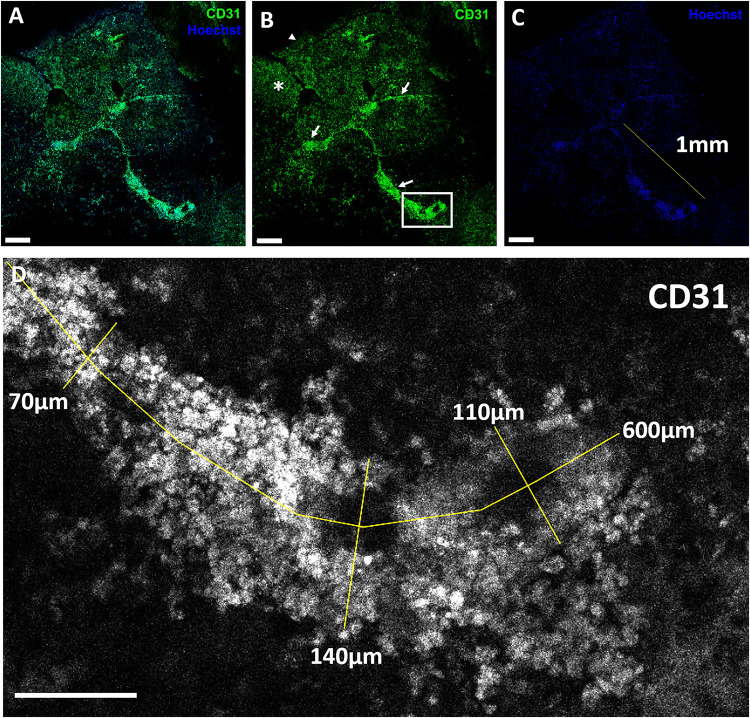
CD31-positive endothelial cell organisation within a 3D bioprinted patch containing HCAECs and HDFs. **(A–C)**. Collapsed Z-stacks of confocal images of a 3D bioprinted AlgGel patch stained with antibodies against CD31 (green) and Hoechst stain (blue). **(A)** Merged channel image of CD31^+^ endothelial cells (green) and nuclei (blue) within a patch fragment. **(B)** CD31^+^ endothelial cells (white arrowhead shows border of hydrogel fragment and white asterisk shows hydrogel containing CD31^+^ cells). A small T-shaped formation of endothelial cells is starting to organise, with small 1 mm branch-like formations starting to form (white arrows). **(C)** The cell nuclei show the same branched organisation which maps to the CD31^+^ endothelial cells in **(B)**. Magnification bars **(A–C)** = 200 μm. **(D)** Magnified black and white image taken from inset panel in **(B)** shows CD31^+^ endothelial cells with measurements (yellow lines, measurements indicated on image; scale bar 100 μm) and this structure is shown in more detail in the 3D rendering shown in Figure 6 and Supplementary Video 2.

**FIGURE 6:**
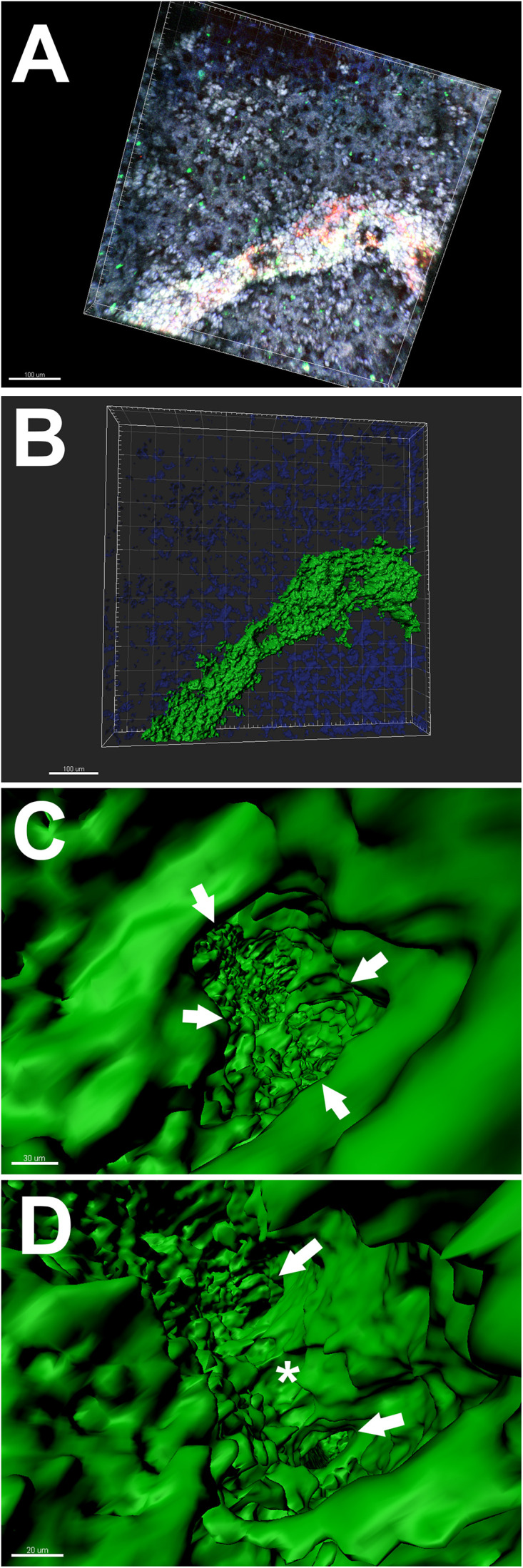
Lumen evaluation of CD31^+^ endothelial cells within a 3D bioprinted patch generated with alginate 4%/gelatin 8% hydrogel. **(A–D)** 3D rendering analysis using Imaris software of a 3D bioprinted patch stained with antibodies against CD31 (green) and Hoechst stain (blue). **(A)** and **(B)** 3D rendering of the endothelial structure shown previously (in Figure 5). Scale bars 100 μm. **(C)** and **(D)** Higher magnification images depicting the inside of the structural lumen formed by endothelial cells within a 3D bioprinted patch. **(C)** Arrows indicate the inner (luminal) surface of the walls (green). Scale bar 30 μm. **(D)** The star indicates a pillar joining two opposing endothelial surfaces where the lumen branches into two smaller lumens (arrows). Scale bar 20 μm. For a full view of the 3D rendering analysis of the same lumen formed within the 3D bioprinted patch please see Supplementary Video 2.

Conversely, CD31^+^ free (not in spheroid) HCAECs started to organise into structures resembling endothelial cell networks (Figures 5, 6 and Supplementary Video 2). The median length of CD31^+^ linear human endothelial cell structures was 88 μm (IQR 62–114 μm), median width was 37 μm (IQR 29–59 μm) and CD31^+^ endothelial cell covered area in the hydrogel was ∼2.1%. 3D rendering structural analysis revealed a lumen-like space between endothelial surfaces with endothelial cells having the appearance of branched structures (Figure 6 and Supplementary Video 2). Taken together, these findings suggest that (1) AlgGel hydrogels combined with this method permit the structural self-organisation of endothelial cells within bioprinted patches even without additional interventions such as supplementation with angiogenic growth factors and (2) endothelial cells in spheroids with no additional angiogenic factors do not fully migrate out into the surrounding hydrogel for as significant a structural organisation into networks to occur – instead the endothelial cells form an irregular distribution across the patch (Figure 4) and some remain in spheroids (Figure 4D).

## Discussion

3D bioprinting is appealing for biomedical engineering as it can be used to produce uniform tissues, is scalable and automatable (Roche et al., 2020). It is also adaptable for use with different biomaterials and cell types, including iPSC-derived cardiac cells given their potential use for heart regeneration (Roche et al., 2020). Our study using alginate 4% (w/v)/gelatin 8% (w/v) hydrogels and cardiac cells for 3D bioprinting of patches presents a promising approach for cardiac bioengineering. Specifically, supporting our hypothesis, our 3D bioprinted patches showed that it is possible for endothelial cells to self-organise into a structural network. Other studies have previously used differing approaches to describe advances in endothelial cell network assembly (Ong et al., 2017b; Cui et al., 2019; Polley et al., 2020; Xu et al., 2020). We have added to this a 3D rendering of micrographic data that shows endothelial cells self-organised into a structural network with a lumen-like space with our method (Supplementary Video 2).

Additionally, as printability and durability are important determinants of whether the patch survives to allow for cells to organise within it, we identified the printability/durability impacts of several factors (bioprinting system, cellular patch content, number of layers of patch depth, minimisation of movement, addition of AlloECM) which had not previously been assessed. As printability can be measured in terms of resolution (e.g., extruded bioink line width), printing accuracy (the degree to which bioprinted constructs match the intended construct set by the blueprint input into the software), shape fidelity (the ability of bioprinted constructs to maintain shape after deposition) and extrudability (the ease of bioink extrusion/flow) (Fisch et al., 2020; Gillispie et al., 2020), we used 5× 5 gridline patterns instead of patches completely filled with bioink to assess these outcome measures. For the first time in direct comparison, we quantified that printing accuracy was 1.7x higher for AlgGel compared to GelMA with our method which was likely related to GelMA’s less predictable extrudability (we quantified that GelMA complete nozzle blockages occurred on average once every six patches compared to no blockages for AlgGel). For durability, macroscopic disintegration (durability) was defined as patch integrity being unacceptable for transplantation of a whole patch – for example, using our surgical patch transplantation method in a murine model of myocardial infarction (Roche and Gentile, 2020) – as this is a durability indicator of practical relevance to the surgeon transplanting patches for *in vivo* models. As printability and durability are critical for generating patches which are useable for transplantation, we thoroughly evaluated these characteristics. GelMA was used as a durable control as it is an established alternative to AlgGel for cardiac patch bioprinting which is highly durable in culture (Koti et al., 2019). Other studies have numerically described detailed rheological characteristics, including (not limited to) storage modulus, viscosity and extrusion pressures for similar hydrogels (Mondal et al., 2019). Our study provides data on highly practical printability measures (such as print accuracy) to inform hydrogel-related choices for patch culture and subsequent transplantation.

As the effect of the bioprinting systems themselves on our outcome measures was not previously compared, optimal bioprinting conditions with AlgGel hydrogels were tested with three different extrusion bioprinters: two screw-driven extrusion systems (a custom made REGEMAT3D model and the commercially available Rokit *INVIVO*) and one using pneumatic extrusion (a CELLINK BIOX bioprinter) (Supplementary Figure 1). We found that 10 × 10 × 0.4 mm patches – sized for *in vivo* rodent cardiac models (Roche and Gentile, 2020) – can be 3D bioprinted with any system with minimal difference to printability (resolution, printing accuracy, shape fidelity and extrudability) or durability in culture. No matter which system was in use, important parameters influencing the printability of a bioprinted series of patches would have included distance between the bioprinting nozzle tip and the six-well plate surface as well as ambient temperature and hydrogel batch-to-batch variability (see Supplementary Materials). Overall, our results suggest that optimising bioprinting parameters was key to the printability and long durability of patches as opposed to the bioprinting platform used (for example, by optimisation pre-testing to determine the ideal flow rate, nozzle speed and temperature settings which were different for each system to work optimally with our hydrogels). It is known that bioprinting parameters and the concentration of AlgGel hydrogels are established determinants of printability and durability (Mancha Sánchez et al., 2020); our study supports this and also adds the finding that the bioprinting system itself was not a strong influencing factor.

We also compared different hydrogel compositions for durable patches that readily retained their macrostructural shape in culture conditions (Figure 2). AlgGel hydrogels are a suitable choice for experiments that allow time for a degree of tissue maturation in the post-printing phase before the hydrogel disintegrates (Bociaga et al., 2019). By combining our durability data with our time to observation of patch contractility data (contractility was observed to begin between day seven and 13), we are able to propose that transplantation of patches for an *in vivo* model may be optimal just before 14 days in culture. Other studies have reported related durability measures, for instance degradation rate as % weight loss of patches in culture up to 14 days (Bociaga et al., 2019). However, ours is the first to report a more clinically relevant durability measure based on usefulness of the patch for its intended purpose – surgical transplantation with an established method for *in vivo* testing (Roche and Gentile, 2020). Furthermore, we report comprehensive durability data with survival beyond 14 days and present data on three previously unevaluated determinants of durability: layer number (patch depth), minimising movement of the patches and the addition of an exogenous factor (AlloECM). AlloECM is a fibroblast-derived extracellular matrix hydrogel used to mimic properties of the extracellular matrix. We found AlloECM could be added to AlgGel bioprinted patches with no change to printability or patch morphology on day one (when bioprinted). However, they all disintegrated by the next day. Unlike the acellular AlgGel patches which gradually broke into smaller pieces over time but left significant pieces of patch in the well (Supplementary Figure 2), the AlloECM-AlgGel patches were completely disintegrated into pieces of residual hydrogel less than ∼1 mm in diameter. This is the first time durability data have been reported for AlloECM mixed in with AlgGel patches and the finding may have been due to incompatibility between AlloECM and AlgGel hydrogel, including overall pH changes or interference with the ionic crosslinking method used. Conversely, the addition of different cell types within a patch could also alter the microenvironment and therefore affect durability but our data showed there was no major durability difference depending on cell type when we evaluated human and mouse cells in free and spheroid formation, respectively (Figure 2). Overall, we determined that bioprinting platform and cellular content are not strong determinants of durability whereas layer number, minimisation of movement in culture media and the addition of an exogenous factor (AlloECM) were strong determinants of durability.

For the cellular component of patches, we bioprinted some patches with VCS which are microtissue aggregates of mixed cardiac cell types. VCS cultures can be readily adapted for other cell types such as stem cell-derived cells and are generated using a scaffold-free, self-sustainable approach that allows self-assembly and organisation of cells in 3D (Gentile, 2016). Our evaluation of cell viability at 28 days after bioprinting demonstrated that over time both VCS bioink and bioink with freely suspended endothelial cells and fibroblasts could be cultured in bioprinted hydrogel patches (Figures 3 and 4). Accurate and automated quantification of viability for cells embedded in autofluorescent hydrogel patches in 3D is challenging (Noor et al., 2019). Nevertheless, viability of freely suspended cells in bioprinted patches (Figure 3) was estimated at approximately 53–61% at day 28, comparable with previous reports from other studies using extrusion bioprinting of 40–80% (Murphy and Atala, 2014; Fisch et al., 2020). For VCS patches, live cell area/total cell area was estimated at ∼72% with a favourable ratio of live to dead cells (9:1). The inability to accurately quantify live cells in 3D spheroids embedded in a 3D patch limits interpretation of this result without further studies. Future studies would benefit from measuring cell viability in 3D in a more automated way, for instance with automated large specimen, serial confocal microscopy techniques aimed at reducing hydrogel autofluorescence artefact. Nonetheless, throughout our study, cells were viable in AlgGel hydrogels.

All BIO X patches containing mouse VCSs presented contractile activity between day seven and 13 and the contractility was transmitted across the patch (Supplementary Video 1). We reported that the average rate for these patches was 288 beats/min. Other studies have reported a beating rate of approximately 180 beats/min for isolated neonatal mouse cardiomyocytes in 2D culture (Ehler et al., 2013) and 500 beats/min for live adult mice *in vivo* (Mahmoud et al., 2015). For these VCS patches, our confocal images of cells migrating from the VCS into the hydrogel (Figure 4) suggested that at day 28 patches contained both intact VCSs and clusters of cells which had migrated into the hydrogel. The combined presence of cell clusters migrating away from VCSs into the hydrogel and those remaining within VCSs may have been responsible for the generalised contractility observed in these patches (Supplementary Video 1). This might explain why patches did not beat until between one and two weeks after bioprinting, allowing time for some cells to migrate from spheroids and make connections across the patch. Our observed contractile activity stopped and re-started with quiescent non-contractile periods of a few days between. This seemed to be related to disturbing the patches, for example when changing culture medium, which also seemed to be a major factor in promoting macroscopic patch disintegration as well. Nonetheless, patches (or fragments of patches) always resumed contractile activity (all remained contractile on day 28). We were unable to reliably measure the electrochemical discharge which causes cardiomyocyte contraction using our contractility-measuring and pacing system because the patches stopped contracting during the transport to the system platform. This was probably also due to movement disturbance from the transport itself and for this reason our contractility analysis was limited to observance of contractions on video microscopy. Future workflows should minimise patch movement as much as possible, including bioprinting directly onto a surface suitable for electrocardiographic recording and pacing without patch transportation.

Bioengineering of heart tissues requires a vascular network for optimal cell survival and function (Fleming et al., 2010; Roche et al., 2020; Wang et al., 2020) and our observations of stained endothelial cells showed that mouse VCSs did not organise into endothelial network structures to the same extent as freely suspended HCAECs with HDFs (Figures 4–6 and Supplementary Video 2). The endothelial network formation shown in Video 2 is structurally comparable to those reported in some other studies (Noor et al., 2019; Roche et al., 2020), which is noteworthy given our relatively low starting density of endothelial cells (∼2500 cells/mm^3^). One recent study (which reported on endothelial cells derived from induced pluripotent stem cells) reported a density of ∼15000 cells/mm^3^ (Noor et al., 2019), so even with our HCAEC density being six-fold less, a structural network started to form. The first clinical trial for epicardial-transplanted patch repair in humans found that a functional benefit may be conferred to the failing heart even with a low starting density of stem cell derived cardiovascular progenitor cells (410/mm^3^) in large (20 cm^2^) patches (Menasché et al., 2018). Our study supports the notion that significant cell growth and self-organisation can occur in patches from a low starting cell density. For future studies, which may use human stem cell-derived cardiac cells or spheroids, additional factors such as vascular endothelial growth factor (VEGF) could be added to promote endothelial cell organisation and cell migration out of spheroids. Alternatively, a mixture of ‘free’ stem cell-derived endothelial cells and cardiac spheroids could be used. For freely suspended endothelial cells and fibroblasts, we used a ratio of 2:1, respectively. Whilst optimal ratios for various cells in spheroid co-culture have been reported (Noguchi et al., 2016; Polonchuk et al., 2017), the optimal ratio of freely suspended HCAECS and HDFs in AlgGel is not known. The physiological ratio of endothelial cells:fibroblasts in the heart is not universally agreed, but the currently accepted ratio is 4:1 (Pinto et al., 2016; Zhou and Pu William, 2016). In our patches, we doubled the number of fibroblasts relative to endothelial cells (to increase their nourishing/supportive influence) but did not equalise the ratio in case they interfered with endothelial cell self-assembly – as they have previously been shown to interfere with the functioning of other cell types (cardiomyocytes) in equal ratio co-culture (Ong et al., 2017b). Future studies will be needed to determine the optimal ratio of endothelial cells to fibroblasts when freely suspended in hydrogel patches.

Future studies will also be needed to functionally test our self-assembled endothelial cell network. If endothelial cell self-assembly into networks is shown to be a successful approach, it is likely to have advantages over other approaches (such as fabricating artificial moulds and lining them with endothelial cells). Specifically, vascular cells which self-organise following physiological signalling in permissive hydrogel may organise into a hierarchical network of different sized vessels and present a more physiological network without exogenous scaffold material. In summary, both the overall approach and the detailed evaluations in this study pave the way for future studies aimed at myocardial regeneration using cardiac patches.

## Conclusion

This study provides data of high practical relevance to inform bioengineering workflows focused on optimising cardiac patches prior to transplantation. Specifically, we have shown that 3D bioprinted cardiac cells are viable in alginate/gelatin hydrogels for at least 28 days in culture, allowing endothelial cells to self-organise into a network and for patch contractility. Bioprinted cardiac patches in optimised conditions may develop/mature according to physiological signals in the pre-transplant phase even without being significantly coaxed or controlled by additional interventions. Taking into account patch durability, we conclude that an optimal moment to transplant patches after a period of maturation is just before 14 days in culture.

## Data Availability Statement

The datasets presented in this study can be found in online repositories. The names of the repository/repositories and accession number(s) can be found below: Zenodo (CERN, Geneva, Switzerland) repository doi: 10.5281/zenodo.4299230.

## Ethics Statement

The animal study was reviewed and approved by the Animal Ethics Committee at the Northern Sydney Local Health District (project number RESP17/55; 20/04/2017).

## Author Contributions

CR contributed to the conceptualisation, data generation, data curation, data analysis, data visualisation, funding acquisition, investigation, methodology, resources, validation, project administration, writing (original draft), manuscript review, and editing. PS cultured and supplied the mouse cardiac cell spheroids used in these experiments. AA was responsible for the supervision, writing (review), and editing. CJ and MX were responsible for the supervision, manuscript review, and editing. CG contributed to the conceptualisation, data generation, data curation, data analysis, data visualisation, funding acquisition, methodology, project administration, supervision, manuscript review, and editing. All authors contributed to the article and approved the submitted version.

## Conflict of Interest

The authors declare that the research was conducted in the absence of any commercial or financial relationships that could be construed as a potential conflict of interest.

## Supplementary Material

Supplementary Figure 1 3D bioprinting platforms used: a custom-made REGEMAT3D model (left column), the ROKIT *INVIVO* (middle column) and the CELLINK BIO X (right column). Hardware **(A–C)** and software **(D–F)** are shown for these three extrusion-based 3D bioprinting systems. The REGEMAT3D customised **(D)** and *INVIVO* ‘Creator K’ **(E)** software were both accessed on a laptop computer connected to the bioprinter by USB cable and the BIO X software **(F)** was entirely inbuilt on the bioprinter touchscreen.

Supplementary Figure 2 3D bioprinted patch fragmentation. Representative phase images of an alginate 4%/gelatin 8% patch on day one **(A)** that was fragmented in culture and became unsuitable for transplantation by day 28 **(B)**.

Supplementary Video 1 Beating cardiac patches containing mouse vascularised cardiac spheroids in alginate/gelatin hydrogel. The patches shown are oscillating in media with an average rate of 258 beats/min, consistent with mouse cardiac cells. Oscillating patches in media are compared with non-contractile elements such as hydrogel in the well and in the video appendices the appearances of non-contractile patches are shown with and without extrinsic movement applied to the microscopy apparatus.

Supplementary Video 2 Three-dimensional rendering of CD31 + endothelial network-like structure within an alginate/gelatin patch containing HCAECs and HDFs. The structure shown has a lumen space and branches. These confluent CD31 + endothelial cells (shown in green) self-assembled into this structure over 28 days in culture following extrusion 3D bioprinting.
