# Supplementary material for: Printability, Durability, Contractility and Vascular Network Formation in 3D Bioprinted Cardiac Endothelial Cells Using Alginate–Gelatin Hydrogels
Source: Front Bioeng Biotechnol. 2021 Feb 26;9:636257. doi: 10.3389/fbioe.2021.636257 (PMC7968457; doi:10.3389/fbioe.2021.636257)
Supplement: Supplementary Figure 1 — 3D bioprinting platforms used: a custom-made REGEMAT3D model (left column), the ROKIT INVIVO (middle column) and the CELLINK BIO X (right column). Hardware (A–C) and software (D–F) are shown for these three extrusion-based 3D bioprinting systems. The REGEMAT3D customised (D) and INVIVO ‘Creator K’ (E) software were both accessed on a laptop computer connected to the bioprinter by USB cable and the BIO X software (F) was entirely inbuilt on the bioprinter touchscreen. [file Image_1.pdf]

SUPPLEMENTARY FIGURES AND LEGENDS

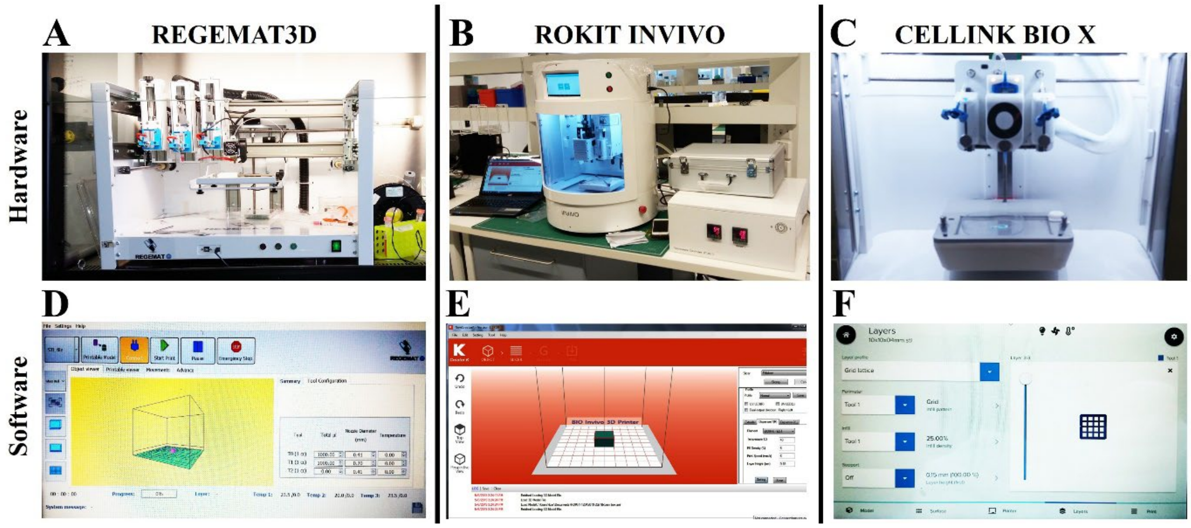

Supplementary Figure 1. 3D bioprinting platforms used: a custom-made REGEMAT3D model (left column), the ROKIT INVIVO (middle column) and the CELLINK BIO X (right column). Hardware (**A-C**) and software (**D-F**) are shown for these three extrusion-based 3D bioprinting systems. The REGEMAT3D customised (**D**) and INVIVO 'Creator K' (**E**) software were both accessed on a laptop computer connected to the bioprinter by USB cable and the BIO X software (**F**) was entirely inbuilt on the bioprinter touchscreen.
