# Supplementary figures and images for: Printability, Durability, Contractility and Vascular Network Formation in 3D Bioprinted Cardiac Endothelial Cells Using Alginate–Gelatin Hydrogels
Source: Front Bioeng Biotechnol. 2021 Feb 26;9:636257. doi: 10.3389/fbioe.2021.636257 (PMC7968457; doi:10.3389/fbioe.2021.636257)

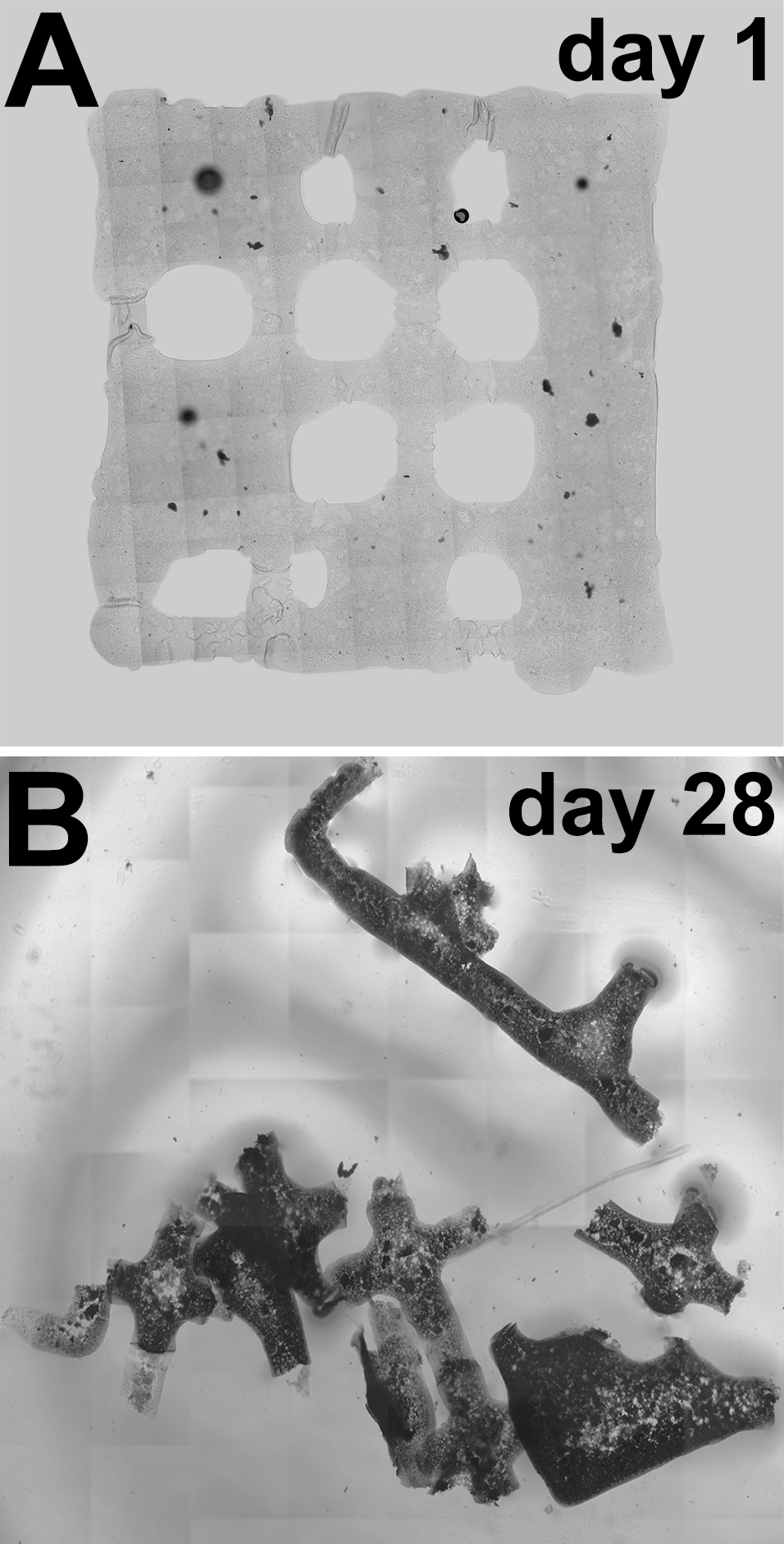

Supplement: Supplementary Figure 2 — 3D bioprinted patch fragmentation. Representative phase images of an alginate 4%/gelatin 8% patch on day one (A) that was fragmented in culture and became unsuitable for transplantation by day 28 (B). [file Image_2.JPEG]
