# Supplementary material for: Printability, Durability, Contractility and Vascular Network Formation in 3D Bioprinted Cardiac Endothelial Cells Using Alginate–Gelatin Hydrogels
Source: Front Bioeng Biotechnol. 2021 Feb 26;9:636257. doi: 10.3389/fbioe.2021.636257 (PMC7968457; doi:10.3389/fbioe.2021.636257)
Supplement: Supplementary file 5 [file Data_Sheet_1.docx]

**SUPPLEMENTARY MATERIALS**

**I. Detailed Bioprinting System Preparation and Parameter Setting Methods**

*Preparation of the 3D Bioprinters*

*Preparation of the REGEMAT3D.* The REGEMAT3D (REGEMAT3D, Granada, Spain) custom-made screw-driven extrusion 3D bioprinter was turned on within a biological safety cabinet pre-sterilised with a germicidal UV lamp. A standard Luer-Lok 3ml syringe was filled with MilliQ water and connected to a Teflon-lined nozzle (200 μm lumen diameter). This is the same nozzle supplied for use with the ROKIT INVIVO bioprinter. The syringe and nozzle were placed, nozzle-down, in 70% (v/v) ethanol and left under a UV lamp for 30 minutes. The syringe was removed and MilliQ water forcibly expelled to clear the nozzle aperture. The syringe was secured in place in the bioprinting mechanism. The bioprinter was calibrated using the REGEMAT3D software platform (full details of all software steps and input parameters available from authors on request).

*Preparation of the INVIVO.* The inbuilt UV lamps and hepafilter of the INVIVO (ROKIT, Seoul, South Korea) commercially-available screw-driven 3D bioprinter allowed for its use on a standard laboratory bench. A 10 ml Leur-Lok syringe was filled with MilliQ water and a Teflon-lined nozzle (200 μm lumen diameter, ROKIT) attached. The syringe and nozzle were placed, nozzle-down, in 70% (v/v) ethanol and left under the UV lamps for 30 mins. The syringe was removed and MilliQ water forcibly expelled to clear the nozzle aperture. The syringe with nozzle and plunger was secured in place in the bioprinting mechanism. The nozzle was calibrated to the printing bed and floor of six well plates using the automated touchpad screen and the software (Creator K), with printing parameters loaded accordingly on a connected laptop computer. Parameters were kept the same throughout all subsequent print runs (key parameters described below, full data available from authors on request). The printer hardware components were set to keep the ambient temperature of the bioprinter chamber between 27.5-28.0°C throughout printing (bed temperature to 37°C; syringe chamber to 39°C). If ambient temperature in the bioprinter chamber exceeded 28°C the chamber was temporarily vented by opening the front panel.

*Preparation of the BIO X.* The inbuilt UV lamps and hepafilter of the BIO X (CELLINK Life Sciences, Boston, MA, USA) commercially-available pneumatically-driven extrusion 3D bioprinter enabled use on a standard laboratory bench. For AlgGel patches, a 3 ml BIO X syringe was filled with MilliQ water and a CELLINK 25-gauge conical polypropylene nozzle (250 μm inner diameter) attached. The syringe and nozzle were placed, nozzle-down, in 70% (v/v) ethanol and left under the UV lamps for 30 mins. The syringe was removed and MilliQ water forcibly expelled to clear the nozzle aperture. The syringe with nozzle was placed in the rightmost (leftmost from operator’s perspective) of the three chambers of the bioprinting mechanism (print head 1 which was chosen for the print run). The nozzle was calibrated manually to the floor of six-well plates using the automated touchpad screen. The printer components were set to the same temperatures as the INVIVO (37°C for the bed and 39°C for the chamber). The ambient temperature was measured using a standard thermometer (although this was less important for the BIO X because print flow rate could be altered during the print run to compensate for ambient temperature-related differences in flow). For ease of transfer of hydrogel to syringe the pneumatic bung between the pneumatic mechanism and the hydrogel was not used (pneumatic force was transmitted directly through air in the syringe to the hydrogel pool at the lowest part of the syringe and in the nozzle). The printing parameters were programmed using the step-by-step touchscreen on the printer (key parameters described below, full data available on request). Initial parameter setting was matched to the INVIVO although for the BIO X, adjustments to flow rate and nozzle travel speed were made during the print runs to create optimal patches based on real-time moment-to-moment operator judgement.

For the gelatin-methacryloyl (GelMA) durability control patches, pre-made light-blocking syringes of 10% w/v + LAP 0.25% in HEPES buffer were purchased (product no. IK305202, CELLINK Life Sciences, Boston, MA, USA) and used without cells. Unlike the AlgGel, these syringes were ready-made and so were loaded directly into the BIO X bioprinter by removing the bung and connecting the syringe chamber to the pneumatic mechanism. For fibroblast-derived extracellular matrix hydrogel (AlloECM®, ROKIT, Seoul, South Korea), 30 mg AlloECM powder was custom-ordered directly from ROKIT, who generated it in a work-intensive proprietary protocol and supplied it for this experiment (the effect of combining AlloECM with AlgGel in our methodology was previously unknown).

*Loading the Bioink.* To transfer the AlgGel-based bioinks to the bioprinting syringes for all bioprinters, the empty syringe chambers without bung or plunger were removed from the bioprinting mechanism by hand. To facilitate transfer of cells in AlgGel, the BIO X syringe’s pneumatic bung was discarded whereas for the REGEMAT3D and INVIVO the syringe plungers were removed and placed head up on the floor of the bioprinter. The syringe chamber was held horizontally, the bioink pipetted into the chamber and syringe plunger replaced (INVIVO and REGEMAT3D). To prevent downward pre-extrusion of hydrogel, the syringe was inverted (nozzle upwards) and plunger advanced to the 1 ml (REGEMAT3D) or 5 ml (INVIVO) position to remove void space in the syringe. The BIO X 3 ml syringe did not require handling of a plunger as syringe chambers connected directly to the pneumatic mechanism and AlgGel did not flow out of the nozzle before bioprinting started. For all bioprinters, the syringes were re-secured, nozzle down, into the bioprinter mechanism. For the INVIVO, re-calibration in the z axis was performed at this point to achieve perfect contact between the nozzle and the six-well plate surface. For the BIO X, re-calibration was not required, calibration was always preserved despite removal, filling and return of the syringe to the bioprinting. The REGEMAT3D required micro-adjustments during bioprinting, by the operator manually twisting the screw-driven extrusion mechanism to increase the flow rate as needed or by manually lifting the plate to make contact with the nozzle.

*Parameter Setting and Extrusion Bioprinting Process*

*AlgGel Bioprinting.* For all bioprinters printing was initiated using previously tested parameters as a GCODE or STL file which had been optimised during preliminary tests. These were not changed for within a bioprinting run. Briefly, patch size was set at 10x10x0.4 mm with two 0.2 ml layers in the z axis and a five-line grid pattern (REGEMAT3D lines were diagonally oriented, INVIVO and BIO X were orthogonally oriented). The bioink flow rate was set at 2 mm/s (REGEMAT3D), 7mm/s (INVIVO) and 6 mm/s (BIO X – initial rate which was changed in real time depending on operator-judged printing outcomes). The grid infill / solidity percentage for the patches was set to produce square grids with five lines of bioink deposited in the x and y axes (**Figure 1, 2** and **Suppl Figure 2**). The infill percentage was set at 6% (INVIVO) and 25% (BIO X) and the equivalent measure of pore size was set at 0.6 mm for the REGEMAT3D. Differences were due to each bioprinter having different software and hardware. Parameters were interlinked, for example, changing the nozzle type on the BIO X software and leaving all other parameters the same changed the appearance of the grid. The travel speed of the REGEMAT3D nozzle across the plate in the x-y axis was 50 mm/sec, for the INVIVO it was 3 mm/s and for the BIO X it was 6 mm/s (for the BIO this was the initial speed which was changed in real time depending on printing outcomes). These were some of the parameters determined at preliminary testing to achieve successful and similar grid-pattern patches, for example, reducing the high speed of the REGEMAT3D resulted in suboptimal bioprinting outcomes. The grid pattern was not always perfectly achieved due to hydrogel lateral flow expansion (non-perfect shape fidelity) after extrusion before crosslinking. For the INVIVO the print bed was designed to optimally hold a four-well square 10 x 10 cm plate and the printer nozzle returned to starting position between each patch being printed; therefore the six well plate we used (which was not square) was manually repositioned before printing the next patch in one of the wells. For all bioprinters we continued until the bioink was depleted or the desired number of successful patches were printed (our range was 4-13 successfully bioprinted patches per bioprinting run and we concluded a bioprinting run after a minimum of one, maximum of three six well plates were used). For six patches 0.5 ml bioink was sufficient if no wastage such as pre-extrusion flow occurred. For the BIO X, recalibration was not necessary as it bioprinted a run of six patches one after the other without interruption and had a one-click recommencement function after changeover of six-well plates (with the same parameters preserved). The BIO X allowed for real-time adjustments during the bioprinting to the nozzle speed and the extrusion pressure which the other bioprinters did not.

*GelMA Bioprinting (with the BIO X).* For the GELMA durability controls, premade GelMA syringes (as described above) were initiated with the slowest settings possible of 1 mm/s print speed and 1 kPa extrusion pressure which were immediately titrated up by the operator once printing began. The flow rate was continuously titrated according to the output seen at the tip of the nozzle to obtain the optimal patch fidelity to a 5 x 5 line two-layer 10 x 10 x 0.4 mm orthogonal grid structure during the print. Significant increases in pressure by the operator were required during the print runs in frequent 2 kPa increments up to ~150 kPa by the end of the bioprinting run of 13 patches. Occasionally, the syringe would block and stop extruding, then due to pressure accumulation, all the GelMA would be expelled and flood the well. At this point the bioprinting was stopped, a new syringe and nozzle at room temperature was loaded and the printing was restarted from the next empty well. The lithium phenyl-2,4,6-trimethylbenzoylphosphinate (LAP) photo-initiator in the GelMA pre-made hydrogel syringes is crosslinked by ultra-violet (UV)-light (instead of ionically by CaCl_2_ - the crosslinking method for AlgGel), so the inbuilt UV curing lights were used to crosslink the patches. These were set to 15 seconds at 100% intensity of 365 nm UV light delivered at the end of each layer being printed at a height of 3 cm between the UV light source and the patch below it. The modular UV curing lights were used rather than the photocuring tool head which did not come as standard with the BIO X unit. After completion of one six-well plate of bioprinted patches, the patches were covered in 3 ml of media per well, the lid was replaced on the plate and it was transferred to the incubator following the same protocol as for AlgGel acellular patches from then on.

**II. Protocols for Fixation, Labelling and Imaging Analysis of Cells**

*Fixation and Immunolabelling of Patches for CD31+ Confocal Imaging.* Following 28 days in culture, bioprinted patches were fixed and stained as follows, with movement/agitation of patches minimised throughout. Patches in six well plates had media aspirated and CaCl_2_ (2% w/v in PBS) added to the well. This was left for 20 minutes at room temperature. For the fixative solution, we mixed 10% (w/v) formalin in neutral buffer (1.2 ml), acetic acid (0.6 ml) and 96% (v/v) ethanol in water (10.2 ml), under a fume hood (final concentrations 1% (w/v) formalin + 9% (v/v) neutral buffer + 5% (v/v) acetic acid + 81.6% (v/v) ethanol + 3.4% (v/v) water). After 20 minutes, the 2% (w/v) CaCl_2_ was aspirated and replaced with the fixative solution described above at room temperature for three hours. Then, the fixative was aspirated and the patch rinsed in PBSA (PBS containing 0.01% sodium azide) for 10 minutes per wash three times. To minimise disintegration, patches were rinsed with 2% (w/v) CaCl_2_ in between PBSA washes. Patches were permeabilised in PBSA containing 0.2% Triton X-100 for 20 minutes at room temperature, then blocked in 3% bovine serum albumin (BSA) in PBSA (w/v; Blocking Solution) for 30 minutes at room temperature. For human cells, mouse anti human primary antibodies against CD31 (product no. 555446. BD Pharmingen™, NJ, USA) were added diluted in Blocking Solution (antibody concentration 50 µl/ml) and incubated in the dark at 4°C overnight. Patches were then washed three times (10 minutes each wash) at room temperature in PBSA with CaCl_2_ rinsing in between each wash. Secondary antibodies (Alexa Fluor® 647 AffiniPure Donkey Anti-Mouse; 715-605-151. Jackson Immunoresearch Laboratories PA, USA) were diluted in blocking solution (7 µl secondary antibody in 1000 µl Blocking Solution). Hoechst stain was added to the same solution (1 drop per 1 ml). This solution was pipetted onto the patch and left for 1.5 hours at room temperature. For mouse cells we fixed and immunolabelled using the same procedures described above except the primary antibody was rat anti-mouse CD31 (553370. BD Pharmingen™, NJ, USA) and secondary antibody was donkey anti-rat (Alexa Fluor® 647 AffiniPure; 712-605-153. Jackson Immunoresearch Laboratories PA, USA). At the end of secondary antibody and Hoechst staining, mouse and human cultures were then washed 3 x 10 minutes in PBSA at room temperature. Patches were mounted onto a microscope glass slide, preserving the 3D depth by adding glass spacers on the side of the patches, the mounting media (VectaShield®, Vector Laboratories, Burlingame, CA, USA), then a coverslip on top and sealing the prepared slide with a film forming polymer (nail varnish) applied to all edges.

*Post-processing for Visualisation of Microscopic Images.* Levels were thresholded with brightness and contrast adjusted in Adobe Photoshop.

*Quantification methods.* For random grid sampling with manual counting of cells, the number of cells per 500 μm^2^ of one layer of patch was manually counted over ten random areas of patch and the average was used to estimate live cell density for one layer which was doubled to account for the two layer depth of the patch. For software-based estimation of live and dead cells, FIJI (ImageJ) software was used to analyse the particles from a thresholded black and white binary image for each of the colour channels, green, red and blue. The total area covered by pixels for the green channel (live cells) or the red channel (dead cells) was divided by the blue channel area (total nuclei) to obtain the estimated ratios of live and dead cells. For length and width measurements of CD31+ endothelial cells within a patch, 20 random measurements were taken of linear CD31+ endothelial cell structures using Adobe Photoshop software and the median and interquartile ranges calculated.

**III. Supplementary Discussion Points:**

*Additional Considerations for Bioprinting System Optimisation*

*Maintaining Optimal Nozzle-plate Contact.* The BIO X allowed for continuous printing into six-well plates after one calibration without losing contact with the plate surface. The INVIVO, which has a 10 x 10 cm printing bed which would fit a four-well plate or a petri dish, required manual rotation of the six-well plate to align the next well to receive the next patch to be printed. This could alter the plate-nozzle distance sufficiently to have a marked effect on the resulting patch. The nozzle-plate distance could become too wide and therefore more hydrogel needed to be extruded before contact with the plate was made. If it was too narrow, the Teflon tip of the nozzle would be squashed into the plate and mechanically disrupt the print layers in the Z axis. In the latter case, the printing of the second layer in the Z axis could be severely disturbed as the height of each of our layers was 0.2 mm and if the nozzle was too deep for the first layer, it would not raise far enough to be clear for the second layer and therefore the Teflon would run through and damage the integrity of the first layer.

*Temperature and Realtime Adjustments.* The BIO X allowed for real time adjustments of flow rate based on the operator’s judgement of how well the bioink was flowing on any given print run to compensate for temperature-related changes in flow. The BIO X also allowed temperature control of the print bed and the syringe-holder but not the ambient temperature in the printing chamber which was not displayed. The INVIVO also does not allow for direct ambient temperature control of the chamber but does display the ambient temperature. Both these bioprinters allowed for control of the temperature of the bioprinting bed and the syringe chambers of the bioprinting mechanism. We found these two temperature settings could be set up initially to achieve predictable warming or cooling of the ambient temperature. Venting the chamber by opening the front panel was used if ambient temperature went above and the optimal temperature which we found to be 28°C for our hydrogel/method. Micro-adjustments could be made during the printing, such as turning down the temperature of the bed once printing begins (as far as zero degrees) so that the patches hit the cold surface of the six-well plate and the hydrogel is less likely to lose its structure. The REGEMAT3D did not allow for any temperature controls. For all three bioprinters, parameters such as flow rates of the bioink out of the nozzle or speed of movement of the nozzle across the plate were set to achieve precise control of the patch morphology (**Figure 1** and **2**). However, an increase or decrease in ambient temperature by even 0.5°C could result in the next patch being markedly different despite all other parameters being the same: too cold and the hydrogel became more viscous and less hydrogel was extruded, too warm and it became less viscous and more hydrogel was extruded.

Overall, the bioprinting parameters (and especially temperature) determined the quality of the patches at baseline on day zero. Some were controllable (such as input software parameters) and others were not controllable (such as humidity or batch-batch hydrogel variability). These parameters seemed to be the major determinant of patch durability. Variation in uncontrollable parameters may explain the variation in durability between patches over the 28 day period despite keeping all controllable parameters the same. Each bioprinter introduced different levels of parameter variation: the BIO X was fully automated (preserving controllable parameters, for example by continuous printing without interruption of patches in six-well plates and one-touch restarting for the print process with existing parameters). However, it also allowed for changes of bioprinting parameters (such as flow rate) in real time during a live print. This allowed the operator to compensate for variability at the time of bioprinting. The INVIVO was similarly automated although printing occurred one patch at a time with the printer performing a re-homing (calibration) cycle before the next patch. As the print bed was designed for a four-well (square) 10 x 10 cm plate, six-well plates had to be manually rotated into position between each patch print, introducing some variability in parameters (such as nozzle-plate distance). The custom-made REGEMAT3D required the operator to insert hands into the print space and make manual adjustments to flow rate and bioprinter bed height throughout the bioprinting process, introducing the most uncontrollable parameter variability of the three bioprinters. Nonetheless, the overall durability of patches was not found to be significantly different between the three bioprinting systems.
